# Understanding Acupoint Sensitization: A Narrative Review on Phenomena, Potential Mechanism, and Clinical Application

**DOI:** 10.1155/2019/6064358

**Published:** 2019-08-14

**Authors:** Huijuan Tan, Steve Tumilty, Cathy Chapple, Lizhou Liu, Suzanne McDonough, Haiyan Yin, Shuguang Yu, G. David Baxter

**Affiliations:** ^1^Centre for Health, Activity and Rehabilitation Research, School of Physiotherapy, University of Otago, Dunedin, New Zealand; ^2^Centre for Health and Rehabilitation Technologies, Institute of Nursing & Health Research, School of Health Sciences, Ulster University, Belfast, UK; ^3^Department of Acupuncture and Tuina, Chengdu University of Traditional Chinese Medicine, Chengdu, Sichuan, China

## Abstract

As part of traditional Chinese medicine, acupoints are considered a dynamic functional area, which can reflect the internal condition of the body. When the body is suffering from disease or injury, corresponding acupoints are believed to be activated and manifest in several sensitized forms, including expansion of the receptive field, pain sensitization, and heat sensitization. Such phenomena are believed to gradually disappear concomitantly with recovery from the disease. Acupoint states are therefore changeable according to health status, a phenomenon known as acupoint sensitization. This review aims to provide an overview of acupoint sensitization based on existing research results and determine priorities for future research. Systematic literature retrieval was conducted in Medline, Embase, Cochrane Library, CINAHL, and AMED from inception to 18 July 2018. Current evidence from research findings to date indicate that acupoint sensitization is based on neurogenic inflammation and that stimulation of sensitized acupoints presents a potential trend of generating a better clinical effect when compared with stimulation of unsensitized points.

## 1. Introduction

As described in the classical acupuncture monograph *Miraculous Pivot* [1], the acupoint is the basis of acupuncture: it can, on one hand, reflect the internal condition of the body; on the other hand, it can be chosen to treat disease.

Regional anatomy studies have shown no intrinsic difference in structure between routine acupoints and adjacent areas [2–4], indicating acupoints represent functional areas, rather than having specific defining structures. Zhou and Benharash argued that the acupoint is a neurogenic point which can exist anywhere on the body as long as there is a sensory nerve [5]. Furthermore, it has been found that when the body is under pathological stress (injury or disease), there are changes in associated acupoints, including sensation threshold and biophysical properties [6–9]. Based on these findings, the concept of “acupoint sensitization” has been proposed: this proposes that the status of acupoints are dynamic and can change from a “silent” to “active” state during pathological processes. Several studies have demonstrated the existence of acupoint sensitization, and this theory has been accepted and refined by an increasing number of scholars. Review of the acupuncture classics indicates that “acupoint sensitization” is referred to as original state of the acupoint (the pathological reaction points were used as acupuncture points in ancient times before any systematic theory of meridian and acupoints being proposed and completed), in line with the account in *The Yellow Emperor's Canon of Internal Medicine* (it is a supplement of the classical acupuncture theory) [8–12].

Acupuncture has been used to treat disease for thousands of years, and selecting routine points on related meridians is widely accepted as a foundational principle in treatment. However, results of systematic reviews and well-designed randomized controlled trials on acupuncture are variable: some support the effectiveness of acupuncture, while others indicate inconclusive clinical benefits when compared with sham treatment [13–17]. Interestingly, it has been well reported that nonacupoint stimulation has a significant “placebo effect” on relieving pain and nausea [18, 19]. A meta-analysis and narrative review suggested that in chronic pain management, acupoint selection (whether fixed or individualized acupoints or nonacupoints) is not associated with acupuncture's effect size [20, 21], suggesting that merely selecting routine acupoints may not be a key feature of acupuncture effects.

Dry needling may be regarded as a simplified version of acupuncture that targets needling of trigger points, which are regarded as a form of pain-sensitized points (i.e., pain threshold at the points is reduced) [22]. Systematic reviews have reported that such dry needling has benefit in several pathological states [23–25]. Furthermore, one review (which analyzed 3728 acupuncture clinical trials) showed better effectiveness of treatment of pain-sensitized points than stimulation of “extra points” and routine acupoints [6]. Liu et al. suggested dry needling is superior to conventional acupuncture for patients with low back pain [26]; it is also reported that the distribution pattern of pain sensitivity of acupoints is notably similar to needle sensation distribution [27]. However, the presence and intensity of needle sensation were not found to be related to better pain relief in patients with osteoarthritis [28]. Selection of points is a central component of acupuncture and moxibustion treatment, but it is unclear whether stimulating sensitized acupoints is superior to routine acupuncture and moxibustion. Thus, it is necessary to assess the clinical relevance of such sensitized points and to provide better evidence for clinical application.

We reviewed the existing literature to address the following questions:

(1) What is the cause and manifestation of sensitized acupoints? (2) What is the mechanism behind this phenomenon? (3) Is stimulation of sensitized acupoints more effective than conventional acupuncture treatment using only routine points (refers to 361 regular acupoints on 14 meridians)?

Our findings are expected to provide preliminary information for future studies.

## 2. Methods

The research questions above were combined into two key focus areas, and search strategies developed accordingly:

1. The phenomena and mechanism of acupoint sensitization
2. The effect of stimulating sensitized points

Systematic searches were performed in Medline, Embase, Cochrane Library, CINAHL, and AMED from database inception to 18 July 2018 using keywords related to “acupoint” and “sensitization” (the detailed search strategy is displayed in Appendix 1 in Supplementary Materials). Reference lists of pertinent articles were reviewed, and related articles and citations were traced in Scopus for supplementary search.

The articles were selected based on following inclusion and exclusion criteria (see Table 1).

## 3. Results

172 articles were obtained as the evidence, which covered both laboratory-based studies and clinical trials. These evidence presented the current research progress on the cause, phenomena, and mechanism of acupoint sensitization and, furthermore, clinical effect of stimulating sensitized acupoints.

### 3.1. Causes of Acupoint Sensitization

While internal pathological factors represent an essential prerequisite for acupoint sensitization, it is important to recognize that an external stimulus (like needling and moxibustion) also plays a role in acupoint sensitization as well [6]. Furthermore, the degree of sensitization is influenced by both disease severity and the intensity of external stimulation. It is reported that more serious diseases are associated with lower pain threshold [29, 30]. Wang [31] gave mustard oil of different concentrations (5%, 7.5%, 10%, 12.5%, 15%, and 17.5%) to rats anally to induce acute intestinal mucosal injury models; results revealed that the number of extravasated Evans Blue (EB) dye points on the skin (the usual criterion to identify sensitized points in animal experiments) were correlated with the concentrations of mustard oil, with the most obvious reactions in the highest concentration group. A relationship between acupoint sensitization and external stimulation was also found when acupoint *Laogong* (PC8) was irradiated by laser (wavelength 808 nm) with different powers (20 mW, 50 mW, and 100 mW), after which optic reflectivity of the acupoint decreased with the increase of laser power [32].

### 3.2. Acupoint Sensitization Phenomena

#### 3.2.1. Reduced Sensation Threshold of Acupoints

Reduced sensation threshold of acupoints is the most common manifestation of sensitized acupoints [8]. Disease-related acupoints may manifest as being sensitive to pain and heat [29, 30, 33–39], with even a slight stimulation inducing an amplified feeling. It was found that the rate of occurrence of heat sensitization increased from 10% to 70% in the presence of conditions such as allergic rhinitis, trigeminal neuralgia, and asthma [37].

Distribution of sensitized acupoints varies. Some are located in the pathological local areas: in patients with gastric ulcer, pressure-pain threshold (PPT) was found to be decreased significantly on *Burong* (ST19) and *Liangmen* (ST21), which are located on the anterior abdomen [29]. It was further reported that pain- and heat-sensitized acupoints were also located in distal areas along the corresponding meridians [30, 35, 40, 41] or bilateral sides of the spine in diseases like allergic rhinitis, lumbar disc herniation, and asthma [38, 39, 42]. Beyond this, some sensitized acupoints have been reported as extra acupoints (they are not on the 14 recognized meridians but have specific names, locations, related diseases, and clinical usage) under certain conditions. These include *Neixiyan* and *Xiyan* for knee osteoarthritis, *Dannang* for cholecystitis, or *Lanwei* for appendicitis [43–45]. Additionally, reduced PPT at auricular acupoints has been reported to reflect pathological states [46–49]: PPT was found to be significantly reduced in auricular acupoints “*heart*” and “*endocrine*” in patients with metabolic syndrome [46]. Interestingly, some points with reduced PPT were also detected on the contralateral side of the body [50–54], strongly suggesting central sensitization mechanisms.

#### 3.2.2. Changes in Biophysical Properties at Acupoints

The biophysical properties of acupoints have also been reported to change in pathological conditions: this includes changes in temperature, electrical, acoustic, and light properties. Increases in temperature around acupoints have been found in a range of conditions as diverse as cholecystitis, gastrointestinal dysfunction, overactive bladder syndrome, intracranial hypertension syndrome, facial paralysis, and mammary gland hyperplasia [55–62]; furthermore, the degree of sensitization could also be aggravated by external stimulus [63–66]. Electrophysiologically, acupoints demonstrate high conductance, i.e., low impedance and resistance [67–69]. However, in the presence of disease, the electrical properties of acupoints change: decreased conductance [70] as well as increased impedance [71] and resistance [72] are reported in stroke, asthma, and dysmenorrhea patients, respectively. However, findings are not always consistent, with Suen et al. [46] showing that auricular acupoint “endocrine” demonstrated a significantly higher conductance in patients with metabolic syndrome. Acupoints' acoustic properties also change in illness [73] so that frequency and amplitude of sound waves were found to reduce in patients with mental illness and functional dyspepsia [74, 75]. Beyond this, it has been reported that there are differences in luminescence intensity and infrared radiation spectrum at acupoints between patients and healthy participants [76–82].

#### 3.2.3. Enlarged Receptive Field of Acupoints

The functional size of acupoints is changeable, and acupoints' receptive fields have been shown to expand during illness. In rats with visceral pain associated with colorectal distension, a visceral nociceptive stimulus caused enlargement of receptive fields of acupoints [83]. In intestinal cancer patients, it was found that PPT at nonacupoints located 1-2 cun (cun is a traditional metric unit used to find acupoint. It is a bone proportional measurement; the width of people's thumb is normally regarded as 1 cun) lateral to acupoints was decreased compared to that of a control group [40], providing additional evidence of expansion of receptive fields. Receptive fields have also been shown to expand after receiving external stimulation. In Latremoliere and Li's studies, after stimulating receptive fields, it was found that further stimulation to nonreceptive fields (3-4 cm away from receptive fields) could also activate neuronal responses [84, 85]. It was also indicated that peripheral receptive fields of convergent neurons were enlarged, which was regarded as external manifestation of central sensitization. Findings are not consistent, however, Chae et al. found that PPT at *Sanyinjiao* (SP 6) in premenstrual syndrome patients was significantly lower than that in healthy controls, but adjacent nonacupoints (2 cm anterior to SP 6) did not show any obvious specificity [30].

#### 3.2.4. Change of Acupoints Morphology

It has been reported that hardened subcutaneous tissue in the area of acupoints is likely to be detected in the presence of disease, which is manifested as a nodule or streak of hardened tissue. Some other abnormalities like papula, dimpling, or change of skin color can also appear [49, 86, 87]. These morphological changes are mainly attributed to contraction and adhesion of connective tissue and blood vessels [49, 87].

### 3.3. Potential Mechanism of Acupoint Sensitization

#### 3.3.1. Change of Acupoint Peripheral Environment

Mechanisms of sensitization have been studied using immune-fluorescent labeling of skin biopsy specimens of human-sensitized points to observe the expression of bioactive substances. Results suggest that there are increased expressions of 5-hoxytryptamine (5-HT), histamine (HA), substance P (SP), calcitonin gene-related peptide (CGRP), and TRPV-1 in sensitized points when compared with nonsensitized control sites [6]. Moreover, the aggregation and degranulation of mast cells in sensitized points have been detected in pathological animal models, which were positively correlated with disease severity [88]. It has also been found that expression of tryptase, HA, 5-HT, SP, CGRP, and bradykinin (BK) was increased in sensitized points of animal models [88–90]. Consistently, local histidine, the precursor of histamine, was found to be decreased at sensitized acupoints according to metabolites detection [91]; these biomediators activate receptors in primary sensory neurons and lead to membrane action potentials. Lei et al. [92] found that excited peripheral capsaicin-sensitive afferents played a crucial role in acupoint sensitization, which could intensify the perception of pain and burning sensation. Kim et al. [93] found that acupoint sensitization was associated with neurogenic inflammation, since the location, characteristics, and function of neurogenic inflammatory spots were the same as sensitized acupoints. In addition, increased local blood perfusion was observed by laser speckle imaging at sensitized acupoints in osteoarthritis, which could contribute to the increased temperature, and again, suggesting the potential existence of local inflammation [94].

#### 3.3.2. Involvement of the Central Nervous System

Dorsal column nuclei (DCN) neurons, wide dynamic range (WDR) neurons, and subnucleus reticularis dorsalis (SRD) neurons are important structures of the visceral pain pathway, connecting with the thalamus, cortex, and other rostral neural centers [95–97]. Studies have indicated that these structures are involved in acupoint sensitization. Visceral nociceptive afferents activated by colorectal distension (CRD) lead to the activation of these neurons, resulting in the enlargement of neuron receptive fields and increased neuron discharges [83, 98–100]. Where the body surface of the corresponding nerve segment had already been sensitized, it was found that applying moxibustion or electroacupuncture (with subthreshold stimulation) could further exacerbate the discharge of neurons induced by CRD [83, 98].

Consistent findings of activation have been obtained in the nucleus ventralis posterior lateralis (VPL), an important neural center for somatovisceral relay: its discharge was increased with CRD-induced visceral pain and was further activated by electroacupuncture at acupoints *Zusanli* (ST 36) and *Shangjuxu* (ST 37) [101]. Moreover, c-Fos protein and NR1 receptor, key markers of neuronal excitability [102], were markedly increased in the dorsal horn of spinal cord and rostral ventromedia medulla (RVM) after CRD [103], highlighting the hyperexcitability in visceral reaction neurons under pathological conditions.

Using functional imaging techniques, a significant difference was found in the pain default mode network (“default mode network” is the network of interacting brain regions that highly correlate with each other. It is active when a participant is awake but not focused on the external environment) between the sensitization and resting states of acupoint *Dubi* (ST35) in knee osteoarthritis patients. It was also found that heat-sensitive moxibustion could specifically regulate central information integration to relieve knee pain, which was considered to be superior to conventional moxibustion [104].

As indicated above, research on mechanism of acupoint sensitization has focused on the local area, spine, medulla, thalamus, and cerebral cortex, respectively; findings have suggested that visceral nociceptive inputs can modulate the reaction of neurons to acupoint stimulation and manifest as increased peripheral neurogenic inflammatory factors, increased neuron discharge, and enlargement of neuron receptive fields. Neurogenic inflammation has generally been regarded as the potential mechanism of acupoint sensitization [8, 90, 93].

### 3.4. Effect of Stimulating Sensitized Acupoints

According to “acupoint sensitization” theory, stimulating sensitized acupoints can easily and effectively activate central excitation to theoretically exert a better effect than stimulation of nonsensitized points [6, 93].

A number of clinical studies have demonstrated the comparative superiority of needle stimulation of pain-sensitized points over conventional acupuncture points, especially in some pain syndromes, where the underlying mechanism might lie in activating endogenous opioid systems [93]. Systematic reviews [105, 106] have demonstrated that for chronic musculoskeletal pain, pain-sensitized point stimulation has advantages over conventional acupuncture, drug therapy, and no treatment. Additionally, a randomized controlled trial found that needling pain-sensitized points could effectively alleviate cervicogenic headache, with more obvious long-term benefit than conventional acupuncture [107]. Small-scale randomized controlled trials have found acupuncturing sensitized points to be more effective than standard acupuncture therapy in patients with chronic low back pain, knee osteoarthritis, or chronic neck pain [108–110]. In addition, superior effects of pain-sensitized points needling has been reported in the management of nonmusculoskeletal diseases like toothache [111], intractable insomnia [112], acute bacillary dysentery [113], migraine [114], and renal colic [115].

Another application of sensitized acupoint stimulation is heat-sensitive moxibustion which is also widely used clinically. This is performed by suspending moxibustion on heat-sensitized acupoints, which will induce a comfortable perception of heat penetration, heat expansion, and heat transmission, as well as sensations like aching, heaviness, pain, and numbness. It is reported that the sensations produced by heat sensitization are closely associated with a better clinical effect [37]. According to a recent systematic review [116], limited evidence supports heat-sensitive moxibustion over conventional moxibustion, acupuncture, and diclofenac sodium in lumbar disc herniation treatment. By contrast, it did not show obvious benefit in bronchial asthma according to another meta-analysis [117]. A multicenter randomized controlled trial [118] suggested heat-sensitive moxibustion worked significantly better than conventional moxibustion and drug injection in knee osteoarthritis. It is also reported that heat-sensitive moxibustion showed superiority over nonsensitive stimulation in easing pain in primary dysmenorrhea patients [119]. Apart from this, it may also improve symptoms like sneezing, running nose, and nasal obstruction in patients with allergic rhinitis when compared with conventional acupuncture [38].

In animal experiments, it is suggested that compared with traditional moxibustion, heat-sensitive moxibustion could more effectively attenuate inflammation and protect ischemic brain site after focal cerebral ischemia/reperfusion injury in rats [120]. Moreover, sensitized acupoints stimulation could significantly improve gastrointestinal function in irritable bowel syndrome, either through electroneedling or moxibustion [121, 122].

## 4. Summary and Future Studies

This review has provided a summary of the evidence on acupoint sensitization: a variety of studies have been completed from different perspectives, using different animal models, different clinical conditions, and different outcome measures. However, while generally in support of the phenomenon of acupoint sensitization, the evidence is mixed. Findings have provided preliminary evidence regarding phenomena and mechanisms of sensitization and potential effectiveness for clinical applications, respectively. Basic studies have confirmed the objective existence of acupoint sensitization, as an external manifestation of dysfunction of internal organs. Mechanistic studies have suggested acupoint sensitization might be caused by the neurogenic inflammation associated with modulation of peripheral substances (like 5-HT, HA, SP, TRPV-1, etc.) and dysfunction of neural activity. Meta-analyses and clinical trials have shown that treatment based on “acupoint sensitization” theory might have more effectiveness than traditional intervention.

However, there are still some issues remaining unresolved. Firstly, based on the evidence to date, the manifestation of acupoint sensitization is diverse and the general criteria still remain unclear; thus, further large-scale epidemiological studies are needed to better characterize the phenomenon. Secondly, as this is a narrative review, we did not perform a quantitative analysis to compare effectiveness between stimulation of sensitized acupoints versus unsensitized acupoints. According to the evidence we obtained, meta-analyses and clinical studies suggested preferable trends in some diseases when stimulating sensitized acupoints either through needling or moxibustion. However, randomized controlled trials on sensitized acupoints stimulation only account for a small proportion of all acupuncture clinical trials. The mainstream research effort still tends to focus on acupoints based on classical acupuncture theory, which recommends use of routine acupoints according to meridian differentiation and syndrome differentiation [123, 124]. Furthermore, systematic reviews find that most studies comparing sensitized acupoint stimulation with conventional acupuncture and moxibustion are of low quality, which makes it hard to draw a definitive conclusion [105, 116]. Therefore, further well-designed, multicenter, large-sample clinical trials are required to confirm the putative benefits of the former method. Thirdly, although changes have been reported in both the peripheral microenvironment and central nervous system, we have little understanding of the complete picture of mechanisms underlying acupoint-sensitized process. This is perhaps not surprising, as acupoint sensitization is a dynamic and complicated process. To address this, dynamic monitoring techniques (such as nanomaterial-functionalized acupuncture needle [125, 126], microdialysis probe [127], etc.) should be used to reveal the whole process of the acupoint's dynamic change. This is expected to provide more evidence on the nature of acupoint and facilitate acupoint selection during acupuncture treatment.

## Figures and Tables

**Table 1:** Inclusion and exclusion criteria of acupoint sensitization studies.

	Inclusion criteria	Exclusion criteria
The phenomena and mechanism of acupoint sensitization	(1) Original research on manifestation or mechanism of acupoint sensitization	(1) Duplicated publications
	(2) Comparing sensitized acupoints with nonsensitized ones on thresholds, biophysical properties, receptive field, morphology	(2) Reviews, letters, comments, editorials, news
	(3) No restriction on study population and language	(3) Research on acupoints in nonpathological condition

The effect of stimulating sensitized points	(1) Original research on clinical effect of acupoint sensitization	(1) Duplicated publications
	(2) Intervention group was stimulation of sensitized acupoints	(2) No controlled group
	(3) Comparison was routine acupuncture or moxibustion	(3) Research on acupoints in nonpathological condition
	(4) Systematic review or controlled trial	
	(5) No restriction on study population, outcome, language	

## Supplementary Materials

Supplementary Materials See Appendix 1 in the Supplementary Material for literature search strategies.
