# Supplementary material for: Understanding Acupoint Sensitization: A Narrative Review on Phenomena, Potential Mechanism, and Clinical Application
Source: Evid Based Complement Alternat Med. 2019 Aug 14;2019:6064358. doi: 10.1155/2019/6064358 (PMC6710800; doi:10.1155/2019/6064358)
Supplement: Supplementary Materials — See Appendix 1 in the Supplementary Material for literature search strategies. [file 6064358.f1.docx]

**Appendix 1**

**Understanding acupoint sensitization: a narrative review**

**Search Strategies**

**Table 1 Database date parameters**

| **Database** | **Dates searched** |
| --- | --- |
| Medline | 1946-18th July 2018 |
| Embase | 1947-18th July 2018 |
| AMED (Allied and Complementary Medicine) | 1985-18th July 2018 |
| CINAHL | 1961-18th July 2018 |
| Cochrane library | CDSR to 2018 Issue 6 of 12  CENTRAL to 2018 Issue 6 of 12 |
| Scopus | Supplementary search |
| Web of Science (WoS) |  |

**Table 2 Database searched**

| **Contents** | | **Databases** |
| --- | --- | --- |
| Q1 | The phenomena and mechanism of acupoint sensitization | Medline/Embase/AMED/CINAHL/Scopus/WoS |
| Q2 | The clinical effectiveness of stimulating sensitized points | Medline/Embase/AMED/CINAHL/ Cochrane library/ Scopus/WoS |

**Q1 The phenomena and mechanism of acupoint sensitization**

**1.1 Medline search terms**

| **A. Acupoint sensitization** | |
| --- | --- |
| A1 | exp Acupuncture Points/ |
| A2 | acupoint*.ab,ti,tw. |
| A3 | exp MERIDIANS/ |
| A4 | or/A1-3 |
| A5 | exp Central Nervous System Sensitization/ |
| A6 | exp PAIN/di, pp [Diagnosis, Physiopathology] |
| A7 | exp Neurogenic Inflammation/ |
| A8 | "sensiti*".ab,ti,tw. |
| A9 | exp Pain Measurement/ |
| A10 | exp Pain Threshold/ |
| A11 | exp hyperalgesia/ |
| A12 | algesia.ab,ti,tw. |
| A13 | infrared.ab,ti,tw. |
| A14 | thermal.ab,ti,tw. |
| A15 | temperature.ab,ti,tw. |
| A16 | exp Electric Conductivity/ or exp Electrophysiology/ |
| A17 | ("electr* propert*" or "electr* conduct*" or "electr* impedance").ab,ti,tw. |
| A18 | exp Acoustics/ |
| A19 | (acoustic propert* or sound wave*).ab,ti,tw. |
| A20 | (light propert* or radiation spectrum).ab,ti,tw. |
| A21 | receptive field.ab,ti,tw. |
| A22 | morphology.ab,ti,tw. |
| A23 | or/A5-22 |
| A24 | A4 and A23 |
| **B. Excluded study designs and publication types** | |
| B1 | letter.pt. |
| B2 | editorial.pt. |
| B3 | news.pt. |
| B4 | comment.pt. |
| B5 | review.pt. |
| B6 | (letter or comment* or review).ti. |
| B7 | or/B1-6 |
| **Combined Search term: A24 not B7** | |
| Result: 1193 | |

**1.2 Embase search terms**

| **A. Acupoint sensitization** | |
| --- | --- |
| A1 | 'acupuncture point'/exp |
| A2 | 'body meridian'/exp |
| A3 | acupoint*:ab,kw,ti |
| A4 | 'acupuncture point*':ab,kw,ti |
| A5 | or/A1-4 |
| A6 | 'central nervous system sensitization'/exp |
| A7 | 'skin sensitization'/exp |
| A8 | 'neurogenic inflammation'/exp |
| A9 | sensiti*:ab,kw,ti |
| A10 | 'pain measurement'/exp |
| A11 | 'pain threshold'/exp |
| A12 | 'hyperalgesia'/exp |
| A13 | 'infrared spectroscopy'/exp |
| A14 | 'infrared radiation'/exp |
| A15 | 'thermal conductivity'/exp |
| A16 | 'temperature sensitivity'/exp |
| A17 | 'skin conductance'/exp |
| A18 | 'skin electrophysiology'/exp |
| A19 | 'acoustics'/exp |
| A20 | 'sound wave'/exp |
| A21 | 'electrical propert*' OR 'acoustic propert*' OR 'light propert*':ab,kw,ti |
| A22 | 'skin receptor'/exp |
| A23 | 'receptive field'/exp |
| A24 | morphology:ab,kw,ti |
| A25 | or/A6-24 |
| A26 | A5 and A25 |
| **B. Excluded study designs and publication types** | |
| B1 | letter OR editorial OR comment OR news OR review:it |
| B2 | letter OR comment* OR review:ti |
| B3 | B1 or B2 |
| **Combined Search term: A26 not B3** | |
| Result: 767 | |

**1.3 CINAHL search terms**

| S1 | MH Acupuncture Points |
| --- | --- |
| S2 | acupoint* OR "acupuncture point*" |
| S3 | MH Meridians |
| S4 | S1 OR S2 OR S3 |
| S5 | "sensiti*" |
| S6 | "Neurogenic Inflammation" |
| S7 | MH Pain Measurement |
| S8 | MH Pain Threshold |
| S9 | MH hyperalgesia |
| S10 | "algesia" |
| S11 | "infrared spectroscopy OR infrared thermography OR infrared thermometer OR infrared radiation" |
| S12 | "thermal" |
| S13 | "temperature n5 increase" |
| S14 | MH Electrophysiology |
| S15 | MH Acoustics |
| S16 | "acoustic propert*" |
| S17 | "sound wave*" OR (MH "Sound Spectrography") |
| S18 | "electr* propert*" |
| S19 | "electr* conduct*" |
| S20 | "electr* impedance" |
| S21 | "light propert*" |
| S22 | "radiation spectrum" |
| S23 | "receptive field" |
| S24 | "morphology" |
| S25 | S5 OR S6 OR S7 OR S8 OR S9 OR S10 OR S11 OR S12 OR S13 OR S14 OR S15 OR S16 OR S17 OR S18 OR S19 OR S20 OR S21 OR S22 OR S23 OR S24 |
| S26 | S4 AND S25 |
| S27 | PT letter OR PT editorial OR PT news OR PT comment OR PT review OR TI letter OR TI comment* OR TI review |
| S28 | S26 NOT S27 |
| Result: 353 | |

**1.4 AMED**

| **A. Acupoint sensitization** | |
| --- | --- |
| A1 | exp Acupoints/ |
| A2 | "acupuncture point*".ti,ab. |
| A3 | exp Meridians/ |
| A4 | or/A1-3 |
| A5 | exp Hypersensitivity/ |
| A6 | "sensiti*".ti,ab. |
| A7 | Neurogenic Inflammation.ti,ab. |
| A8 | exp Pain measurement/ |
| A9 | exp Pain threshold/ |
| A10 | exp Hyperalgesia/ |
| A11 | algesia.ti,ab. |
| A12 | infrared.ti,ab. |
| A13 | thermal.ti,ab. |
| A14 | exp Temperature/ |
| A15 | exp Electrophysiology/ |
| A16 | (electr* propert* or electr* conduct* or electr* impedance).ti,ab. |
| A17 | exp Acoustics/ |
| A18 | (acoustic propert* or sound wave*).ti,ab. |
| A19 | (light propert* or radiation spectrum).ti,ab. |
| A20 | receptive field.ti,ab. |
| A21 | morphology.ti,ab. |
| A22 | or/A5-21 |
| A23 | A4 and A22 |
| **B. Excluded study designs and publication types** | |
| B1 | (letter or editorial or news or comment or review).pt. |
| B2 | (letter or comment* or review).ti. |
| B3 | or/B1-2 |
| **Combined Search term: A23 not B3** | |
| Result: 231 | |

**Q2 The clinical effectiveness of stimulating sensitized points**

**2.1 Medline search terms**

| **A. Stimulation of sensitized points** | |
| --- | --- |
| A1 | exp Acupuncture Points/ |
| A2 | acupoint*.ab,ti,tw. |
| A3 | exp MERIDIANS/ |
| A4 | or/A1-3 |
| A5 | exp Central Nervous System Sensitization/ |
| A6 | exp PAIN/di, pp [Diagnosis, Physiopathology] |
| A7 | exp Neurogenic Inflammation/ |
| A8 | "sensiti*".ab,ti,tw. |
| A9 | or/A5-8 |
| A10 | A4 and A9 |
| A11 | exp Trigger Points/ |
| A12 | (Ashi point* or heat sensiti* point* or tender point*).ab,ti,tw. |
| A13 | or/A10-12 |
| A14 | treatment.mp. or exp Therapeutics/ or intervention.mp. |
| A15 | "stimulat*".ab,ti,tw. |
| A16 | exp ACUPUNCTURE/ or exp ACUPUNCTURE ANALGESIA/ or exp ACUPUNCTURE THERAPY/ or exp ELECTROACUPUNCTURE/ |
| A17 | exp MOXIBUSTION/ |
| A18 | or/A14-17 |
| A19 | A13 and A18 |
| A20 | dry needling.ab,ti,tw. |
| A21 | or/A19-20 |
| **B. Study designs and Publication types** | |
| B1 | exp Meta-Analysis/ |
| B2 | exp Meta-Analysis as Topic/ |
| B3 | (meta analy* or metanaly* or metaanaly*).ab,ti. |
| B4 | "systematic* review*".ab,ti. |
| B5 | randomized controlled trial.pt. |
| B6 | controlled clinical trial.pt. |
| B7 | exp Randomized Controlled Trial/ |
| B8 | exp Randomized Controlled Trials as Topic/ |
| B9 | or/B1-8 |
| **Combined Search term: A21 and B9** | |
| Result: 594 | |

**2.2 Embase search terms**

| **A. Acupoint sensitization** | |
| --- | --- |
| A1 | 'acupuncture point'/exp |
| A2 | 'body meridian'/exp |
| A3 | acupoint*:ab,kw,ti |
| A4 | 'acupuncture point*':ab,kw,ti |
| A5 | or/A1-4 |
| A6 | 'central nervous system sensitization'/exp |
| A7 | 'skin sensitization'/exp |
| A8 | 'neurogenic inflammation'/exp |
| A9 | sensiti*:ti,kw,ab |
| A10 | or/A6-9 |
| A11 | A5 and A10 |
| A12 | 'trigger point'/exp |
| A13 | 'ashi point' OR 'heat sensiti* point*' OR 'tender point*':ab,kw,ti |
| A14 | or/A11-13 |
| A15 | 'therapy'/exp |
| A16 | 'stimulation'/exp |
| A17 | Intervention:ab,kw,ti |
| A18 | 'acupuncture'/exp OR 'acupuncture analgesia'/exp OR 'electroacupuncture'/exp |
| A19 | 'moxibustion'/exp |
| A20 | or/A15-19 |
| A21 | A14 and A20 |
| A22 | 'dry needling':ab,kw,ti |
| A23 | or/A21-22 |
| **B. Study designs and publication types** | |
| B1 | 'meta analysis'/exp OR 'meta analysis (topic)'/exp |
| B2 | 'meta analy*' OR 'metanaly*' OR 'metaanaly*':ab,kw,ti |
| B3 | 'systematic review'/exp OR 'systematic review (topic)'/exp |
| B4 | 'controlled clinical trial'/exp OR 'controlled clinical trial (topic)'/exp |
| B5 | or/B1-4 |
| **Combined Search term: A23 and B5** | |
| Result: 659 | |

**2.3 Cochrane library search terms**

| **A. Stimulation of sensitized points** | |
| --- | --- |
| #1 | MeSH descriptor: [Acupuncture Points] explode all trees |
| #2 | acupoint*:ti,ab,kw (Word variations have been searched) |
| #3 | MeSH descriptor: [Meridians] explode all trees |
| #4 | #1 or #2 or #3 |
| #5 | MeSH descriptor: [Central Nervous System Sensitization] explode all trees |
| #6 | MeSH descriptor: [Pain] this term only |
| #7 | MeSH descriptor: [Neurogenic Inflammation] explode all trees |
| #8 | sensiti*:ti,ab,kw (Word variations have been searched) |
| #9 | #5 or #6 or #7 or #8 |
| #10 | #4 and #9 |
| #11 | MeSH descriptor: [Trigger Points] explode all trees |
| #12 | "heat sensiti* point*":ti,ab,kw (Word variations have been searched) |
| #13 | "Ashi point*" or "tender point*":ti,ab,kw (Word variations have been searched) |
| #14 | #10 or #11 or #12 or #13 |
| #15 | MeSH descriptor: [Therapeutics] explode all trees |
| #16 | treatment or intervention:ti,ab,kw (Word variations have been searched) |
| #17 | stimulat*:ti,ab,kw (Word variations have been searched) |
| #18 | MeSH descriptor: [Acupuncture] explode all trees |
| #19 | MeSH descriptor: [Acupuncture Analgesia] explode all trees |
| #20 | MeSH descriptor: [Acupuncture Therapy] explode all trees |
| #21 | MeSH descriptor: [Electroacupuncture] explode all trees |
| #22 | MeSH descriptor: [Moxibustion] explode all trees |
| #23 | #15 or #16 or #17 or #18 or #19 or #20 or #21 or #22 |
| #24 | #14 and #23 |
| #25 | "dry needling":ti,ab,kw (Word variations have been searched) |
| #26 | #24 or #25 |
| Result: 887 (Review: 19, Trials: 864) | |

**2.4 CINAHL search terms**

| S1 | MH Acupuncture Points |
| --- | --- |
| S2 | acupoint* OR "acupuncture point*" |
| S3 | MH Meridians |
| S4 | S1 OR S2 OR S3 |
| S5 | "sensiti*" |
| S6 | "Neurogenic Inflammation" |
| S7 | S5 OR S6 |
| S8 | S4 AND S7 |
| S9 | MH trigger point |
| S10 | "Ashi point*" OR "tender point*" |
| S11 | "heat sensiti* point*" |
| S12 | S8 OR S9 OR S10 OR S11 |
| S13 | "treatment" OR "intervention" |
| S14 | MH Therapeutics |
| S15 | "stimulat*" |
| S16 | MH acupuncture OR MH acupuncture therapy OR MH electroacupuncture OR MH acupuncture analgesia |
| S17 | MH moxibustion |
| S18 | S13 OR S14 OR S15 OR S16 OR S17 |
| S19 | S12 AND S18 |
| S20 | MH dry needling |
| S21 | S19 OR S20 |
| S22 | (MH "Meta Analysis") |
| S23 | "meta analy*" |
| S24 | "metanaly*" |
| S25 | "metaanaly*" |
| S26 | (MH "Systematic Review") |
| S27 | "systematic* review*" |
| S28 | (MH "Randomized Controlled Trials") |
| S29 | "controlled clinical trial*" |
| S30 | S22 OR S23 OR S24 OR S25 OR S26 OR S27 OR S28 OR S29 OR S29 |
| S32 | S21 AND S30 |
| Result: 154 | |

**2.5 AMED**

| **A. Acupoint sensitization** | |
| --- | --- |
| A1 | exp Acupoints/ |
| A2 | "acupuncture point*".ti,ab. |
| A3 | exp Meridians/ |
| A4 | or/A1-3 |
| A5 | exp Hypersensitivity/ |
| A6 | "sensiti*".ti,ab. |
| A7 | Neurogenic Inflammation.ti,ab. |
| A8 | 5 or 6 or 7 |
| A9 | 4 and 8 |
| A10 | "trigger point*".ti,ab. |
| A11 | "Ashi point*".ti,ab. |
| A12 | "heat sensiti* point*".ti,ab. |
| A13 | "tender point*".ti,ab. |
| A14 | 9 or 10 or 11 or 12 or 13 |
| A15 | exp Therapy/ |
| A16 | (stimulat* or treatment or intervention).ti,ab. |
| A17 | exp Acupuncture/ |
| A18 | exp Electroacupuncture/ or exp Acupuncture analgesia/ |
| A19 | exp Acupuncture therapy/ |
| A20 | exp Moxibustion/ |
| A21 | 15 or 16 or 17 or 18 or 19 or 20 |
| A22 | 14 and 21 |
| A23 | dry needling.ti,ab. |
| A24 | 22 or 23 |
| **B. Study designs and publication types** | |
| B1 | exp Meta analysis/ |
| B2 | (meta analy* or metanaly* or metaanaly*).ti,ab. |
| B3 | "systematic* review*".ti,ab. |
| B4 | controlled clinical trial.pt. |
| B5 | randomized controlled trial.pt. |
| B6 | exp Randomized controlled trials/ |
| B7 | or/B1-6 |
| **Combined Search term: A24 and B7** | |
| Result: 108 | |
